# Taxonomic Characterization and Secondary Metabolite Profiling of *Aspergillus* Section *Aspergillus* Contaminating Feeds and Feedstuffs

**DOI:** 10.3390/toxins7093512

**Published:** 2015-09-02

**Authors:** Mariana Greco, Minna Kemppainen, Graciela Pose, Alejandro Pardo

**Affiliations:** 1 Laboratorio de Micología Molecular, Departamento de Ciencia y Tecnología, Universidad Nacional de Quilmes, Bernal 1876, Argentina; E-Mails: minnakemppainen90@gmail.com (M.K.); apardo@unq.edu.ar (A.P.); 2 Consejo Nacional de Investigaciones Científicas y Técnicas, Buenos Aires 1033, Argentina; 3 Escuela de Producción, Tecnología y Medio Ambiente, Universidad Nacional de Río Negro, Villa Regina 8336, Argentina; E-Mail: npose@unrn.edu.ar

**Keywords:** animal feed spoilage, xerophilic species, teleomorphs, mycotoxins, scanning electron microscopy

## Abstract

Xerophilic fungal species of the genus *Aspergillus* are economically highly relevant due to their ability to grow on low water activity substrates causing spoilage of stored goods and animal feeds. These fungi can synthesize a variety of secondary metabolites, many of which show animal toxicity, creating a health risk for food production animals and to humans as final consumers, respectively. Animal feeds used for rabbit, chinchilla and rainbow trout production in Argentina were analysed for the presence of xerophilic *Aspergillus* section *Aspergillus* species. High isolation frequencies (>60%) were detected in all the studied rabbit and chinchilla feeds, while the rainbow trout feeds showed lower fungal charge (25%). These section *Aspergillus* contaminations comprised predominantly five taxa. Twenty isolates were subjected to taxonomic characterization using both ascospore SEM micromorphology and two independent DNA loci sequencing. The secondary metabolite profiles of the isolates were determined qualitatively by HPLC-MS. All the isolates produced neoechinulin A, 17 isolates were positive for cladosporin and echinulin, and 18 were positive for neoechinulin B. Physcion and preechinulin were detected in a minor proportion of the isolates. This is the first report describing the detailed species composition and the secondary metabolite profiles of *Aspergillus* section *Aspergillus* contaminating animal feeds.

## 1. Introduction

Fungal contamination of foods and feeds causes negative effects on the quality of the products mainly reducing their nutritional and organoleptic properties and lead consequently to important annual economic losses worldwide [1,2,3,4]. Moreover, fungi are capable of elaborating a wide range of secondary metabolites many of which have been shown to be highly toxic. Therefore, fungal contamination creates a serious health threat for animals as well as for humans. *Aspergillus* and its teleomorphs are important mycotoxin producers and these fungi constitute an important contaminant of cereals and feedstuffs [5,6,7,8]. Especially, *Aspergillus* species of the section *Aspergillus*—members of the teleomorphic genus *Eurotium* in the previous nomenclature [8]—have been reported to be able to contaminate a variety of biological materials of low water activity. These include stored grains and cereals [9], dried salted fish [10], bakery products [11], mixed feeds and raw materials [5], cultural assets and old books [12,13], and even human corpses [14]. *Aspergillus* section *Aspergillus* species have also been isolated from hypersaline waters of salterns [7], air samples near buildings [15] and from the Dead Sea [16].

Section *Aspergillus* species are generally considered benign fungi for human health and they are used in oriental food fermentation processes as starter cultures [8]. Species of this section have been isolated and also new species described from natural fermentation products, such as meju, a dried fermented soybean paste typically consumed in Korea [17,18,19]. Although generally considered free of mycotoxins, some of the secondary metabolites produced by the section *Aspergillus* fungi are known to show antioxidative, antibacterial, antifungal and antiprotozoal activities [20,21,22]. Some of these compounds, such as echinulin, physcion and flavoglaucin, even though not considered mycotoxins *sensu stricto*, do show toxicity to animals [23,24,25,26]. In addition, the production of more potent mycotoxins such as aflatoxins, gliotoxin, sterigmatocystin and ochratoxin, by the *Aspergillus* section *Aspergillus* species is controversial [9,27,28,29,30,31,32,33]. Several published reports of such mycotoxic potential exist, but these studies have been criticized for lacking further confirming studies using taxonomically properly characterized fungal isolates, or no repetitive mycotoxin detection has been achieved [9,27,28,29,30,31,32,33]. Therefore, the true toxicogenic capacity of *Aspergillus* section *Aspergillus* requires revision. In addition, the growth of xerophilic fungi, like the section *Aspergillus*, results in release of metabolic water. This increases the water activity of the contaminated materials in time. Such change in the physico-chemical properties of the substrates can permit the growth of other less xerophilic and highly toxicogenic fungi, such as *Alternaria*, *Penicillium* and *Fusarium*, and consequently result in further contamination of foods and feeds with even more potent mycotoxins.

The mycotoxin-contaminated animal feeds do not have potentially adverse effects only on animal health and productivity but they can cause further secondary contaminations of human consumers via eggs, meat, or milk [34,35]. *Aspergillus* section *Aspergillus* teleomorphs have been isolated from rabbit and chinchilla feeds as well as from poultry feeds with very high frequencies in Argentina [36,37]. More importantly, these studies have demonstrated that the given animal feeds were also contaminated with various potent mycotoxins (aflatoxins, deoxynivalenol, fumonisins, ochratoxin A, T2-toxin and zearalenone). Even though the detected toxin levels were lower than the regulation limits established, these contaminations with multiple mycotoxins were simultaneous and affected up to 80%–100% of the studied samples. Because the section *Aspergillus* fungi can produce multiple secondary metabolites with adverse effects on animal health and also reports of even more hazardous mycotoxins production exist, these high contamination levels are suggesting that both a direct and an indirect risk for animal and human health could be linked to the use of these contaminated animal feeds. This makes the further studies on the degree of xerophilic fungal contamination, the detailed species composition identification and especially, the toxicological characterization of the isolates obtained from animal feeds very valuable. Access to such information would help to evaluate the true mycotoxicological risk generated by the use of feeds in animal production in Argentina. Moreover, fungal strains isolated from different matrices, habitats and geographical origins are expected to have different metabolite profiles and toxicogenic potentials [38]. Therefore, local information is needed as the results obtained from animal feeds used in other geographical areas cannot be directly extrapolated.

The aim of the work presented here was to study in detail the degree of xerophilic fungal contamination in variable commercial and non-commercial animal feedstuff and primary raw materials destined to rabbit, chinchilla, and rainbow trout production in Argentina. The *Aspergillus* section *Aspergillus* species composition was thoroughly characterized and the production of the secondary metabolites was profiled in 20 isolates belonging to the dominant feedstuff contaminating section *Aspergillus* species. Especially, the evaluation of the secondary metabolite production capacity was expected to clarify the currently controversial position of section *Aspergillus* species as a toxin free taxon.

The *Aspergillus* section *Aspergillus* isolates obtained in this study and the previous bibliographic data were treated according to the current nomenclature rules of the Code of Botanical Nomenclature for Algae, Fungi and Plants [39] and the International Commission on *Penicillium* and *Aspergillus* (ICPA) [40]. The isolated teleomorphic fungal states are referred by their genus *Aspergillus* species names. This recently proposed change in the fungal nomenclature has also faced criticism as the introduction of the teleomorphic genus *Eurotium* into the genus *Aspergillus*, with the consequent changes of the species nomenclature, is feared to cause widespread confusion [41]. To avoid any confusion to the readers, the five *Aspergillus* section *Aspergillus* species most relevant to this study and supported by the recent taxon revision by Hubka and colleagues [42] are presented in Table 1, together with their corresponding former genus *Eurotium* names.

**Table 1:** The actual nomenclature of five *Aspergillus* section *Aspergillus* species. The taxa in the teleomophic genus *Eurotium* have been transferred to the genus *Aspergillus*, according to the one-species-one name principle, and the *Eurotium* names should thus not be used anymore. The teleomorph of *A. proliferans* was described after this nomenclature change [42].

Previous Nomenclature	Current Nomenclature
*Eurotium amstelodami*	*Aspergillus montevidensis*
*E. chevalieri*	*A. chevalieri*
*E. herbariorum*	*A. glaucus*
*E. repens*	*A. pseudoglaucus*
*E. rubrum*	*A. ruber*
*---*	*A. proliferans*

## 2. Results 

### 2.1. Taxonomic Identification of Section Aspergillus Species Based on Growth and Ascospore Characteristics

The presence of xerophilic fungi of *Aspergillus* section *Aspergillus* was analysed in commercial and non-commercial producer assembled formulations of rabbit, chinchilla, and rainbow trout feeds used in animal production in Argentina. The fungal contamination of animal feeds can happen both during the pre- and post-fabrication phases. Neither do all the producers in the region use commercial animal feed formulations for animal feeding. Therefore, the presence of xerophilic fungi was also tested in a wide variety of primary raw materials used either for direct feeding or for preparation of homemade animal feed mixes by the producers (alfalfa pellets, wheat, corn and soybean derivatives, and bone and meat flour).

The initial isolation of section *Aspergillus* species was carried out on DG18 medium, a standard selective medium used for enumeration and isolation of xerophilic fungi from dry and semi-dry foods and feeds. Twenty-one samples of rabbit feeds, 25 samples of chinchilla feeds, 28 samples of rainbow trout feeds and one sample of each of the nine raw material types were analysed for their fungal charge leading to isolation of altogether 522 putative section *Aspergillus* isolates. The morphological species level identification was done according to the taxonomic key of Pitt and Hocking [8], focused on identification of five teleomorphic species dominant as contaminants of low water activity substrates worldwide. This identification resulted, in the first place, in the detection of four section *Aspergillus* species among the isolates: *A. montevidensis*, *A. chevalieri*, *A. pseudoglaucus* and *A. ruber*. Only a minor number of isolates could not be identified using the given taxonomic key. The total isolation frequency (Fr%) of *Aspergillus* section *Aspergillus* spp. and the Fr% of the four identified species isolated from the different animal feeds and the primary raw materials are presented in Table 2. Elevated total isolation frequencies (>60%) were obtained from both the rabbit and chinchilla feeds while the fungal charge of the studied rainbow trout feeds was somewhat lower (25%). The four identified *Aspergillus* species were present both in rabbit and chinchilla feeds. In the case of rainbow trout feeds, only *A. pseudoglaucus* and *A. ruber* were initially detected. However, further growth morphological analyses suggested that all the isolates initially identified as *A. ruber* from the trout feeds were in fact *A. proliferans* and that this fifth species was also present in the rabbit and chinchilla feeds analysed (see below). Among the primary raw materials, pelleted alfalfa, wheat bran, wheat millrun, pelleted soy and corn seeds all tested positive for section *Aspergillus* fungi. No isolates were obtained from meat and bone flower, inactivated soy or commercially produced whole grain and milled corn.

Three species of section *Aspergillus*; *A. ruber*, *A. proliferans* and *A. glaucus*, are known to show very similar colony morphology on CYA, MEA and CY20S media. In addition, their general ascospore characteristics are relatively similar and these species can therefore easily be confused with each other. Due to their close resemblance, some authors have treated *A. ruber* and *A. glaucus* as synonyms or *A. glaucus* and *A. proliferans* as a single species [43,44,45,46]. Recent taxonomic analyses using both micro- and macromorphological growth characteristics, physiological data, DNA fingerprinting and four independent DNA loci sequences, however, strongly support the independent evolutionary identities of these three species [42]. This study also described the teleomorph of *A. proliferans*, the only species in the section *Aspergillus* without a link between its ana- and teleomorphic states.

**Table 2:** Total isolation frequencies (Fr%) of *Aspergillus* section *Aspergillus* spp. and those of the four identified species from the animal feeds and the primary raw materials analysed. The Fr% of the unidentified section *Aspergillus* spp. includes isolates which could not be identified with certainty based on their growth morphological characteristics according to the identification key of Pitt and Hocking [8]. The proportion of *A. proliferans* among *A. ruber* isolates was estimated based on their restricted growth on CY20S [42].

Animal Feed/ Raw Material	Fr%					
	Total	*A. montevidensis*	*A. chevalieri*	*A. pseudoglaucus*	*A. ruber* (*A. proliferans*)	Unidentified *Aspergillus* spp.
Rabbit feed *	61.9	19.1	23.8	33.4	14.3 (1/7)	4.8
Chinchilla feed **	60	44	12	12	16 (5/7)	12
Rainbow trout feed ***	25	ND	ND	17.9	14.3 (7/7)	3.6
Pelleted alfalfa (p)	100	100	ND	ND	100 ^+^	ND
Wheat bran (p)	100	ND	100	ND	ND	ND
Wheat millrun (c)	100	100	ND	ND	ND	ND
Pelleted soybeans (p)	100	ND	100	100^+^	ND	DN
Corn seeds (p)	100	ND	ND	ND	ND	100

* data based on 21 analysed samples presenting three different feed formulations, ** data based on 25 analysed samples presenting three different feed formulations, *** data based on 28 analysed samples presenting two different feed formulations. One sample of each raw material type was analysed. (p) producer’s own preparation, (c) commercial product. ND, not detected. ^+^ The initial isolate identification was corrected after SEM and DNA analyses.

According to the taxonomic key of Hubka *et al.* [42] *A. glaucus* can be distinguished from the two other species by its predominantly larger ascopores (6–7.5(8.5) µm), while *A. ruber* is able to grow on M60Y medium at 37 °C, a characteristic not shared by *A. proliferans*. Also, the growth of *A. proliferans* is significantly more restricted on CY20S medium at 25 °C after seven days (<30 mm) than the growth of *A. ruber* or *A. glaucus* (>30 mm). Unfortunately, the information of this different growing capacity on M60Y medium as a fundamental tool for discrimination between *A. ruber* and *A. proliferans* was not available during the experimental set up of the present study. Nevertheless, the evaluation of the obtained *A. ruber* isolates for their ascospore sizes suggested total exclusion of *A. glaucus*. Even though relative high size variation could be detected even within individual isolates, the mean ascospore sizes were closer to 5 than 6 µm and no isolates with larger ascopores, characteristic for *A. glaucus*, were detected. On the other hand, the growth responses on CY20S medium were indicating a heterogeneous nature for the initial *A. ruber* isolate collection. These could be divided into two marked growth response groups; one with maximum up to 30 mm diameter colonies (*i.e.*, *A. proliferans*) and one with significantly vigorous colony growth (>40–50 mm), characteristic for *A. ruber*. This suggested the presence of *A. proliferans* as a fifth section *Aspergillus* species isolated from the starter materials. Later, the DNA sequence data further confirmed this growth morphological identification of *A. proliferans* (see below) allowing a rough estimation of the presence of *A. proliferans* among the isolates initially identified as *A. ruber* (Table 2).

The taxonomic characterization was continued with 20 isolates including representatives of four growth morphologically identified species, *A. montevidensis*, *A. chevalieri*, *A. pseudoglaucus* and *A. ruber*. This set of isolates consisted of five representatives of the first three section *Aspergillus* species and four of *A. ruber*. In addition, one putative isolate of *A. proliferans*, identified by its restrictive growth response on CY20S medium, was included for confirming this growth morphological detection of the species. The isolates originated from all three types of animal feed mixes studied and from pelleted soy and alfalfa used as primary raw materials. The 20 isolates were subjected to further electron microscopic analysis, DNA level taxonomic characterization and secondary metabolite profiling.

### 2.2. Scanning Electron Microscopy (SEM)

**Figure 1:**
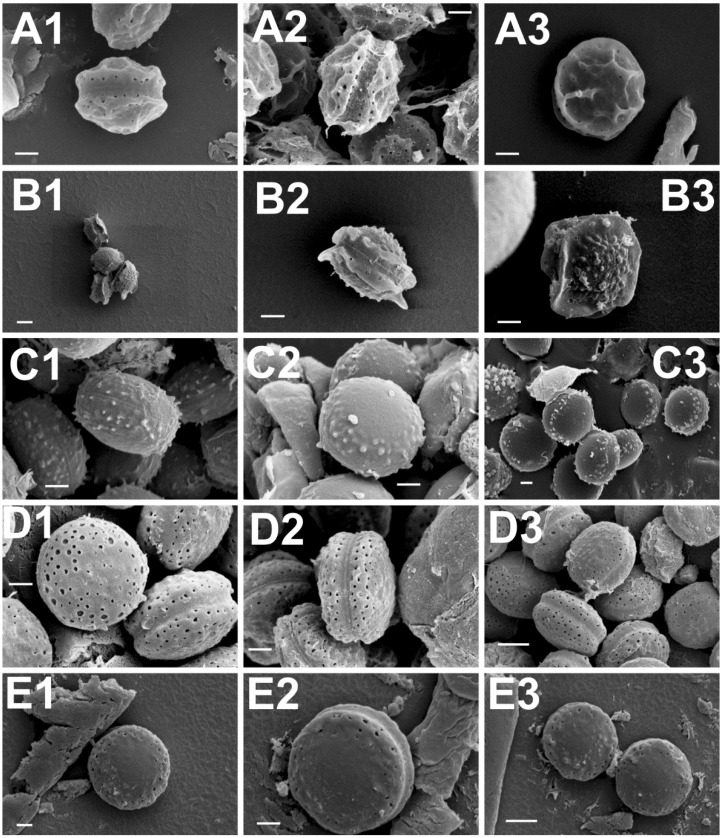
Scanning electron microscopy photos of ascospores of *A. montevidensis* (**A**, 1–3); *A. chevalieri* (**B**, 1–3); *A. pseudoglaucus* (**C**, 1–3); *A. ruber* (**D**, 1–3) and *A. proliferans* (**E**, 1–3). Scale bars: A(1–3)-B(2,3)-C(1–3)-D(1–3)-E(1,3) = 1 µm; B1, E2 = 2 µm.

Scanning electron microscopy (SEM) has traditionally been used as a tool for species classification in the section *Aspergillus*. Especially ascospore size, surface ornamentation and features of the equatorial region have offered characteristics of taxonomic value for species differentiation. To further confirm the species identity of the selected isolates, their ascospore micromorphology was analysed by SEM. The results were concordant with the growth morphological species identification and confirmed the isolates as representatives of the respective four *Aspergillus* section *Aspergillus* species. The ascospore characteristics did not significantly differ from those already described [8,47]. A set of representative SEM photos are shown in Figure 1 and a summary of the ascospores characteristics is presented in Table 3. Two isolates, originally identified based on the growth characters and light microcopy observation of the ascospores as *A. montevidensis*, were re-identified as *A. chevalieri* by SEM. In addition, one isolate originally considered as *A. chevalieri* was re-identified as *A. ruber*. These changes in the isolate species identity were further supported by DNA analyses (see below) and demonstrates the value of SEM as a complementary tool for species identification in the section *Aspergillus*. It is however noteworthy that the ascospores of *A. proliferans* and *A. ruber* have highly similar size and micromorphology and therefore SEM does not offer solid separation of these two species. Moreover, some strains of *A. ruber* are reported to show atypical ascospore morphology making spore size and surface ornamentation very weak identification characteristics for *A. ruber* and *A. proliferans* [42]. Our SEM study resulted in detection of slightly bigger and smoother surfaced ascospores in the case of the studied *A. proliferans* isolate; otherwise, the general morphological characteristics and the size of the ascopores between *A. proliferans* and *A. ruber* were highly similar. These results confirm the weak taxonomic resolution of SEM in separating *A. ruber* from *A. proliferans*, a goal that, besides some growth characteristics, can solidly be reached by DNA analyses.

**Table 3:** Ascospore sizes (µm) and the micromorphological characteristics of the five *Aspergillus* section *Aspergillus* species isolated from feedstuffs.

Species	Ascospore Measurements (µm) and Ornamentation				
	Size	Surface	Furrow	Pores	Ridges
*A. montevidensis*	4.2–4.5	reticulated	pronounced	0.1	short
*A. chevalieri*	3.8–4.6	small peaks	shallow	0.1–0.2	long, wavy
*A. pseudoglaucus*	4.2–4.6	small peaks	minimum	absent	absent
*A. ruber*	4.6–5.8	protuberances	pronounced	0.1–0.2	absent
*A. proliferans*	5.2–5.3	smooth	pronounced	0.1–0.3	absent

### 2.3. Taxonomic Identification Based on DNA Analyses

The final confirmation of species identity of the 20 isolates was obtained through PCR amplification and sequencing of the nuclear ribosomal ITS1-5.8SrDNA-ITS2 region and beta-tubulin gene fragment. The recent combined phylogenetic analysis of Hubka and colleagues [42], based on three independent protein encoding DNA loci (beta-tubulin, calmodulin and RNA polymerase domain 2), has postulated recognition of altogether 17 *Aspergillus* section *Aspergillus* species. Fourteen of them are located within three major clades, each of which with strongly supported terminal clades corresponding to independent species of the section. *Aspergillus glaucus* clade includes closely related species of *A. glaucus* and *A. proliferans*, *A. niveoglaucus*, *A. brunneus* and *A. neocarnoyi* while *A. ruber* clade consists of *A. ruber*, *A. pseudoglaucus*, *A. appendiculatus* and *A. tonophilus*. The *A. chevalieri* clade is formed by *A. chevalieri*, *A. montevidensis*, *A. intermedius*, *A. cristatus* and *A. costiformis*. The only species located outside of these three major clades are *A. cibarius*, *A. xerophilus* and *A. leucocarpus* [42]. Posterior to Hubka’s analyses also two new species, *A. cumulatus* and *A. osmophilus* have been described from rice straw used in meju fermentation in Korea [19] and from contaminated cereals in Iran [48]. Then, a study conducted by Visagie *et al.* [49] described the new species *A. sloanii* on *Aspergillus* section *Aspergillus* isolated from indoor house dust in United Kingdom. The morphological characteristics and DNA data indicates that these are true new evolutionary species reaching a total of 20 species in *Aspergillus* section *Aspergillus*.

The phylogenetic analyses of the 20 studied isolates were conducted using the type-strain reference sequences of altogether 18 currently described section *Aspergillus* species. The accession numbers of the studied isolates are presented in Table 4. The species, strain numbers and sequence of the type-strain reference and more detailed information of the previous nomenclature are available in Supplementary Materials (Table A1 and Table A2, respectively). In the case of the four species, *A. montevidensis*, *A. chevalieri*, *A. pseudoglaucus* and *A. ruber*, not only the type-strain sequences but also non-type strains were included for increasing the sequence diversity. *Aspergillus* *osmophilus* was excluded from the analyses due to its very large ascospore size (8.5–10 µm), a characteristic which was not detected among our 20 isolates. At the DNA marker level, this species is most related to *A. xerophilus* and does not form part of the three major terminal multispecies clades of the section [48]. The proposed novel species, *A. cumulatus*, was however included as its ascospore characteristics overlap with the studied isolates [19].

In order to confirm the presence of *A. proliferans* among the studied isolates, special attention was paid to this taxon. The recently described *A. proliferans* teleomorph is from many growth morphological characteristics highly similar to *A. ruber*, while at the DNA maker level it is very close to its sister species, *A. glaucus* [42]. As being frequently misidentified in the past, the true global species abundance and the intraspecies sequence variation of *A. proliferans* is not well known yet. Neither has the teleomorph of this species been described in Argentina before. In order to increase the diversity of the geographical origin of the isolates, not only the *A. proliferans* type-strain, isolated from United Kingdom, but also non-type isolates from United States and Czech Republic and two fungal isolates, described as novel *A. proliferans* teleomorphs from Iran [48], were included in the analyses.

**Figure 2:**
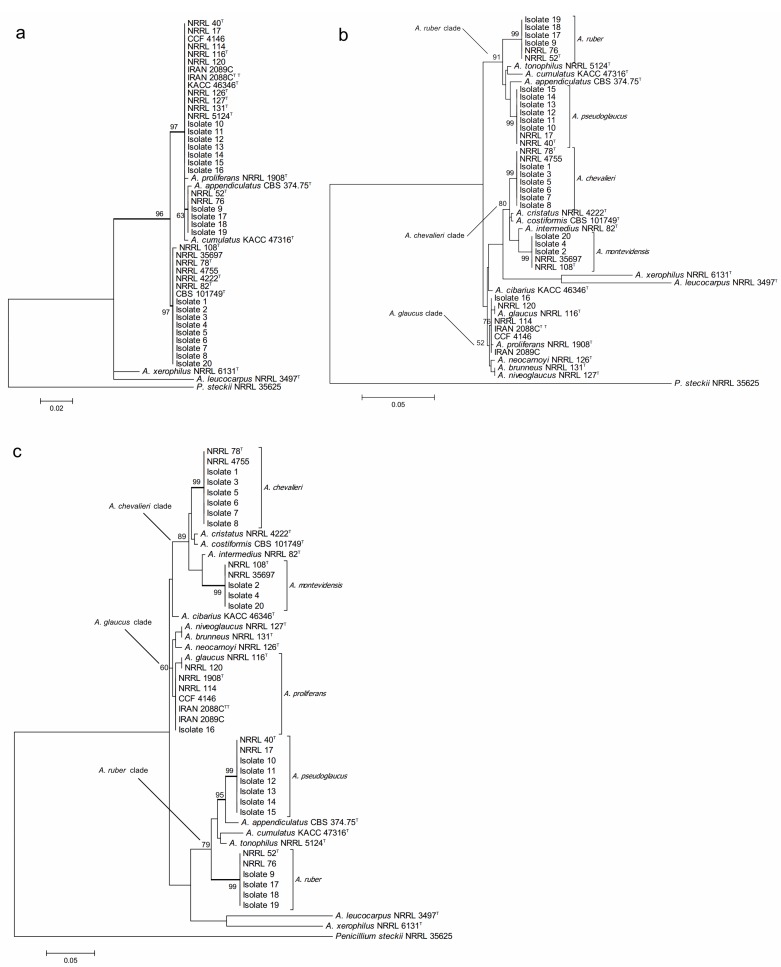
Phylogenetic trees generated by Maximum Likelihood analysis showing the relationship of 18 type strains of *Aspergillus* section *Aspergillus* species and the 20 isolates obtained from animal feeds and primary raw materials. Three based on (**a**) the ITS region; (**b**) the beta-tubulin gene fragment; and (**c**) the concatenated analysis of both. *Penicillium steckii* (NRRL 35625) was used as outgroup. The thickened lines represent lineages with >90% bootstrap values. The Bootstrap analyses were performed with 1000 replications. ^T^ Ex-type strain ^TT^ Ex-teleotype strain.

The maximum likelihood analysis based on the ITS sequences (Figure 2a) resulted in very weak resolution of the *Aspergillus* section *Aspergillus* at species level. All the 20 studied isolates located within two major multi-species terminal clades with strong bootstrap support but none of them could be identified at species level. One of the clades included both the 11 studied isolates and all the species of *A. glaucus* and *A. ruber* clades, and *A. cibarius*. Within this multispecies clade, four of the studied isolates formed part of a moderately supported internal subclade. However, this subclade was neither conspecific nor included both *A. ruber* and *A. appendiculatus*. The second major multispecies clade consisted of the rest of the nine studied isolates and the species known to belong to the *A. chevalieri* clade of the section.

**Table 4:** ITS and beta-tubulin sequences of the isolates under study.

Species Name	Isolate	GenBank Accession Number	
		ITS	Beta Tubulin
*A. montevidensis*	2	KT373923	KT373942
*A. montevidensis*	4	KT373925	KT373944
*A. montevidensis*	20	KT373941	KT373960
*A. chevalieri*	1	KT373921	KT373922
*A. chevalieri*	3	KT373924	KT373943
*A. chevalieri*	5	KT373926	KT373945
*A. chevalieri*	6	KT373927	KT373946
*A. chevalieri*	7	KT373928	KT373947
*A. chevalieri*	8	KT373929	KT373948
*A. proliferans*	16	KT373937	KT373956
*A. pseudoglaucus*	10	KT373931	KT373950
*A. pseudoglaucus*	11	KT373932	KT373951
*A. pseudoglaucus*	12	KT373933	KT373952
*A. pseudoglaucus*	13	KT373934	KT373953
*A. pseudoglaucus*	14	KT373935	KT373954
*A. pseudoglaucus*	15	KT373936	KT373955
*A. ruber*	9	KT373930	KT373949
*A. ruber*	17	KT373938	KT373957
*A. ruber*	18	KT373939	KT373958
*A. ruber*	19	KT373940	KT373959

On the other hand, the phylogenetic analysis based on the beta-tubulin encoding gene fragment resulted in discrimination between altogether 17 of the 18 *Aspergillus* section *Aspergillus* species included in the study. Only *A. niveoglaucus* and *A. brunneus* could not be separated from each other with this DNA marker. The study also successfully confirmed the species identity of all the studied isolates (Figure 2b). The growth morphological species identification with the additional SEM data was confirmed and the studied isolates were identified as three isolates of *Aspergillus montevidesis*, six of *A. chevalieri*, six of *A. pseudoglaucus*, four of *A. ruber* and one as *A. proliferans*. The morphological identification of *A. proliferans* from *A. ruber* based on its restricted growth on CY20S medium was confirmed as the given isolate (isolate 16) located together with the four other *A. proliferans* sequences used in the analysis. The species identity of only one of the isolates, considered *A. ruber* before DNA analysis changed to *A. pseudoglaucus*.

The sequences of the 20 studied isolates were in all the cases 100% identical to GenBank type-strain reference sequences. The three characteristic multispecies clades of the section (*A. chevalieri*, *A. glaucus* and *A. ruber* clades) were clearly detectable and all the terminal species clades with studied isolate sequences were strongly supported having bootstrap values higher than 80.

A further concatenated phylogenetic study was run joining the ITS and b-tubulin sequences data (Figure 2c). As expected, this analysis did not change the species identity of the studied isolates but it did slightly modify the bootstrap support of the three major clades and some of the terminal clades as well. In most of the cases, the support of the nodes was reduced. Joining the two DNA loci data did not result in separation between *A. niveoglaucus* and *A. brunneus* either, demonstrating that the maximum species level discrimination for *Aspergillus* section *Aspergillus* was reached already with one protein encoding DNA locus, the b-tubulin fragment.

### 2.4. Secondary Metabolite Profiles

**Figure 3:**
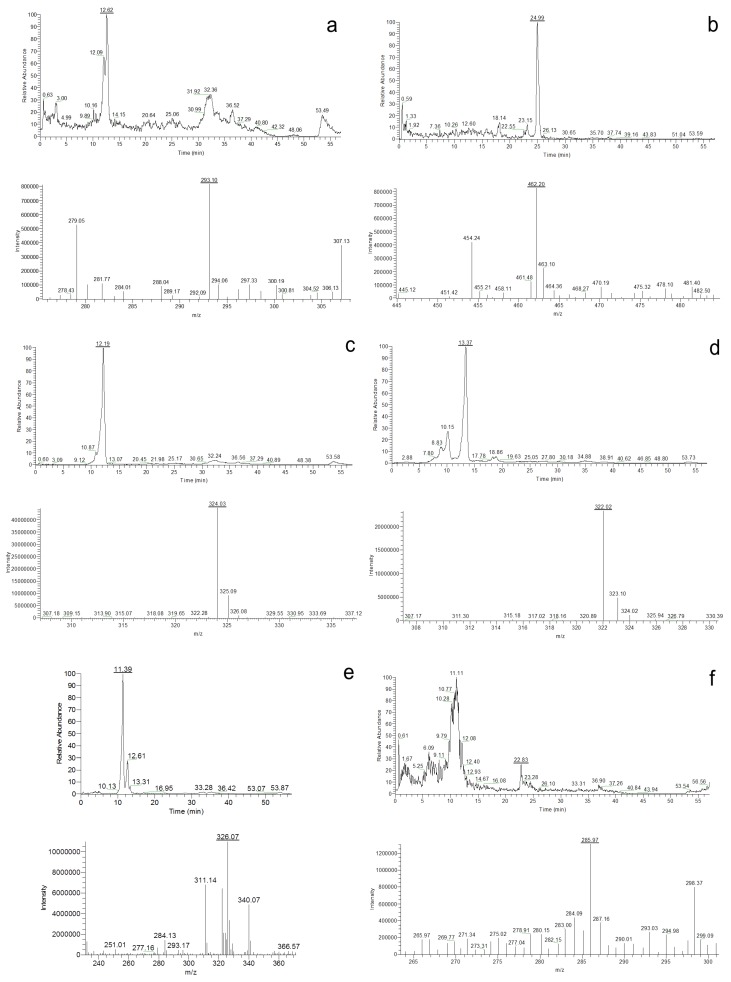
Chromatograms and ESI spectrum of (**a**) Cladosporin (*m/z* 293); (**b**) Echinulin (*m/z* 462); (**c**) Neoechinulin A (*m/z* 324); (**d**) Neoechinulin B (*m/z* 322); (**e**) Preechinulin (*m/z* 326); (**f**) Physcion (*m/z* 285).

Once the species identity was comprehensively established for the 20 isolates, their secondary metabolite profiles were analysed. The HPLC-MS analysis showed the presence of cladosporin, echinulin, and neoechinulin A and B (Table 5). All the studied isolates, representatives of the five species of section *Aspergillus*, were able to produce neoechinulin A. Eighteen isolates produced neoechinulin B, and 17 isolates were positive for cladosporin and echinulin. In addition, preechinulin and physcion were also detected. The Figure 3 shows representative chromatograms and the ESI spectrum of the determined secondary metabolites. None of the species showed a fixed profile, but a moderate intraspecies variation in the secondary metabolite production was detected.

**Table 5:** Secondary metabolites of the five *Aspergillus* section *Aspergillus* species isolated from feeds and feedstuffs (ND: Not Detected).

Species	Secondary Metabolite					
	Cladosporin	Echinulin	Neoechinulin A	Neoechinulin B	Preechinulin	Physcion
*A. montevidensis*	+	+	+	+	+	ND
*A. montevidensis*	+	+	+	+	ND	ND
*A. montevidensis*	+	+	+	+	ND	ND
*A. chevalieri*	+	ND	+	+	+	ND
*A. chevalieri*	+	+	+	+	ND	+
*A. chevalieri*	+	+	+	+	ND	+
*A. chevalieri*	+	+	+	+	ND	ND
*A. chevalieri*	+	+	+	+	ND	ND
*A. chevalieri*	+	+	+	+	ND	ND
*A. pseudoglaucus*	+	+	+	+	ND	ND
*A. pseudoglaucus*	+	+	+	+	ND	ND
*A. pseudoglaucus*	+	+	+	+	ND	ND
*A. pseudoglaucus*	ND	+	+	+	ND	ND
*A. pseudoglaucus*	+	ND	+	ND	ND	ND
*A. pseudoglaucus*	+	ND	+	+	ND	ND
*A. ruber*	ND	+	+	+	ND	ND
*A. ruber*	ND	+	+	+	ND	ND
*A. ruber*	+	+	+	+	ND	ND
*A. ruber*	+	+	+	ND	ND	ND
*A. proliferans*	+	+	+	+	ND	ND

All three *A. montevidensis* isolates were able to produce cladosporin, echinulin, neoechinulin A and B while one of them was also producing preechinulin.

The culture extracts of the six *A. chevalieri* showed presence of cladosporin and neoechinulin A and B and in only one of the isolates echinulin was not detected. The same *A. chevalieri* isolate was also positive for preechinulin and two other were detected producing physcion.

From the six *A. pseudoglaucus* isolates, three were positive for all four secondary metabolites; cladosporin, echinulin, and neoechinulin A and B. One isolate produced both echinulin and neoechinulin A and B but did not produce cladosporin while the two other isolates were positive for cladosporin, neoechinulin A but negative for echinulin. One of these strains did however test positive for neoechinulin B production.

Four filtrate extracts of *A. ruber* strains were analysed. Among them, one isolate contained cladosporin, echinulin, neoechinulin A and B, another isolate was positive for cladosporin, echinulin and neoechinulin A. The remaining two isolates were positive for echinulin and neoechinulin A and B.

The *Aspergillus proliferans* isolate studied showed production of cladosporin, echinulin, and neoechinulin A and B.

## 3. Discussion 

*Aspergillus* section *Aspergillus* species are found in foods and feeds at all a_w_ levels but they are able to cause spoilage even below 0.90 a_w_ [11]. They are also important secondary metabolite producers and, therefore, the section *Aspergillus* species, previously known as members of the teleomorphic genus *Eurotium*, are considered one of the most destructive xerophilic fungi. Very high isolation frequencies of section *Aspergillus* taxa have been previously reported for rabbit, chinchilla and poultry feeds in Argentina. These feeds have also tested positive for multiple very hazardous mycotoxins [36,37], suggesting that potential mycotoxicological risks could be linked to their use. Consumption of mycotoxin-contaminated feeds can provoke animal illnesses leading to unnecessary suffering and economic losses. In addition, secondary contaminations of humans as final consumers are possible via consumption of contaminated meat and other animal origin products.

Even though generally considered free of potent mycotoxins, section *Aspergillus* fungi are important producers of metabolites with toxic potential [6,7]. They can produce a wide variety of secondary metabolites such as echinulin, neoechinulin A, neoechinulin B, preechinulin, cladosporin, questin and physcion [7,15,50,51]. These compounds have received less attention than more potent mycotoxins, produced predominantly by other fungal species. Therefore, their presence in foods and feeds is not currently controlled or regulated in any country. However, numerous reports exist on the toxic effects of the secondary metabolites produced by section *Aspergillus* species on animals over the last 70 years. According to Cole and Cox [52], some *Aspergillus* species (*A. chevalieri* and *A. montevidensis*) should be considered mycotoxicogenic, and in these cases, echinulin has been the main toxic compound produced. Concordantly, Ali *et al.* [26] have reported liver and lung damages caused by echinulin in rabbits. Furthermore, strains of section *Aspergillus* species contaminating cereal grains have been demonstrated to be toxic for experimental animals, causing lowered weight gains in chickens, toxicity to chicken embryos, dermatoxicity in rabbits, hemorrhaging in chickens, hepatotoxicity in mice, and death of calves, rabbits and mice [53,54,55,56,57,58,59]. In addition, Vesonder *et al.* [59] reported that a feed refused by swine, a situation which led to decreased milk production and consequently to piglets death, was contaminated with echinulin (8 µg/g) and contained high propagule density of *A. montevidensis* and *A. chevalieri*. These fungal isolates were further confirmed to be able to produce echinulin *in vitro* on rice or cracked corn. On the other hand, compounds isolated from *A. montevidensis* and *A. chevalieri* show different activity against malaria, bacteria and cancer cell lines [21,60], metabolites isolated from *A. cristatum* have inhibitory activity on tumour cell lines [61], alkaloids produced by *A. ruber* show anti-oxidant activity [62], and secondary metabolites isolated from *A. pseudoglaucus* (physcion and echinuilin) are cytotoxic to sex cells of the sea urchin *Strongylocentrotus* [63].

In the light of such broad evidence of variable toxic effects of secondary metabolites produced by the section *Aspergillus*, these fungal species can hardly be simply considered benign to animal or human health without further investigation. Variation in the toxicogenic potential between different species and even different isolates are expected to exist. Therefore, in order to evaluate the potential mycotoxicogenical risk linked to section *Aspergillus* contaminations, further studies on the general fungal abundance, species composition and especially on the secondary metabolite profiles of different species and isolates are needed. Such data are not currently available for section *Aspergillus* fungi isolated from foods or animal feeds.

With these means, we have conducted a detailed evaluation of the xerophilic mycobiota present in various commercial and non-commercial feed formulations and primary raw materials used for rabbit, chinchilla and rainbow trout production in Argentina. The isolation trials and the growth morphological species identification confirmed the previous reports [36] of the high contamination frequencies of the rabbit and chinchilla feeds by *Aspergillus* section *Aspergillus* taxa (>60% of the studied samples), while the rainbow trout feeds showed somewhat lower fungal charge (25%). In addition, some of the primary raw materials tested (pelleted alfalfa, soy, wheat bran, wheat millrun and corn seeds) were positive for section *Aspergillus* fungi. These contaminations consisted predominantly of multiple species but the specific species compositions varied both between the feeds and the primary raw material types studied. The presence of five dominant section *Aspergillus* species was identified both in the rabbit and chinchilla feeds (*A. montevidensis*, *A. chevalieri*, *A. pseudoglaucus*, *A. ruber* and *A. proliferans*). However, in chinchilla feed samples *A. montevidensis* was the most abundant taxon, while in rabbit feeds three species; *A. montevidensis*, *A. chevalieri* and *A. pseudoglaucus* were the most frequently isolated ones, led by *A. pseudoglaucus*. On the other hand, rainbow trout feeds showed a completely different species composition formed only by two species, *A. pseudoglaucus* and *A. proliferans*, both with quite similar isolation frequencies. These variations in species abundance and composition most probably reflect different natures and complexities of the primary raw materials used for manufacturing different animal feed formulations, even though possible impacts of post-fabrication storage conditions cannot be excluded. This conclusion was supported further by the isolation results from the primary raw materials studied. When tested positive, fungal contamination was by a single or maximum two section *Aspergillus* species and species compositions were clearly variable between the different types of primary raw material analysed.

The correct and solid species identification is the base of any biological study. The taxonomy and nomenclature of *Aspergillus* section *Aspergillus* species has been under revision during the last years. The transfer of the teleomorphic genus *Eurotium* to the anamorphic *Aspergillus* genus, according to the one-species-one scientific name concept of the botanical code, has led to re-evaluation of the previous data on linkage of anamorphic and teleomorphic fungal states and the species identity. Unfortunate misidentifications of culture collection deposited isolates, contradictory posterior species descriptions and the use of various synonymous species names during the years has seriously complicated this task. However, recently, DNA analyses, together with physiological and growth characters, have been used for clarifying the true taxonomic species position and to solidly link the teleomorphs with their anamorphic states. The studies of Peterson [64] and Hubka *et al.* [42] have profoundly revised the previous species nomenclature and the identity of taxa in the section *Aspergillus*. The growth morphological characters and light microscopical observation of the ascospores, traditionally used for section *Aspergillus* species identification, are to some extent error prone due to their subjectivity. Currently, access to vast molecular taxonomic data from type-strains of section *Aspergillus* species allows a fast and precise direct isolate identification or growth morphological species identity confirmations.

The taxonomic position of the 20 isolates, representatives of the five dominant feedstuff contaminant species of section *Aspergillus: A. montevidensis*, *A. chevalieri*, *A. proliferans*, *A. pseudoglaucus* and *A. ruber*, identified by their growth morphological characters, was confirmed further using both scanning electron microscopy (SEM) and two independent DNA loci. Scanning electron microscopy allows detailed observation and description of ascospore size and structural characteristics, such as surface ornamentation, pores and their arrangement, longitudinal groove and ridges. These have served and still do serve as important characteristics for species identification in the section *Aspergillus* [8,17,42,47,65,66]. However, the ascospore characteristics can also be highly similar between different species, like is the case with *A. ruber* and *A. proliferans*. In addition, a relative high intraspecific and even intraisolate variation of the ascospore characteristics can exist. Therefore, SEM as a species classification and identification tool has a supplementary value, but it is not conclusive. This was confirmed by our SEM study as it allowed to correct some erroneous growth morphological species identifications but did not offer separation between *A. ruber* and *A. proliferans* isolates.

The conclusive confirmation species identity of the isolates was reached with the phylogenetic analysis based on the beta-tubulin gene fragment. The nuclear ITS region has been established as a universal DNA barcode marker for fungi [67]. However, this marker does not necessary have a species level resolution in all fungal taxa. Specifically in the genus *Aspergillus*, identical ITS sequences are known to be shared between several complexes. Therefore, other general protein encoding DNA loci (such as beta-tubulin, calmodulin or RNA polymerase subunit 2) are frequently used for species identification [40,42,64,68]. The degree of ITS species resolution can also vary between different sections of *Aspergillus*. For example, a phylogenetic study based on ITS sequences of the *Aspergillus* section *Circumdati* resulted in identification of 18 out of 27 species [69]. In our phylogenetic analyses of 18 section *Aspergillus* species, the ITS did not allow species level identification for any of them. On the other hand, the analysis based on the beta-tubulin gene fragment led to species level identification for 16 out of 18 of the reference taxa and of all the studied isolates. These results are concordant with reports of beta-tubulin locus as a very potent single DNA marker for species level resolution in *Aspergillus* section *Aspergillus* [17,42,66,70].

High isolation frequencies of section *Aspergillus* taxa were obtained from the animal feeds analysed in the present study similar to earlier reports from Argentina [36,37]. This raises the question of the already existing or potential contamination of such feeds with multiple bioactive and toxic secondary metabolites known to be produced by the section *Aspergillus* fungi. In addition, the evaluation of the potential toxicological risks requires previous knowledge on the toxicogenic capacity of the section *Aspergillus* species contaminating feeds. No previous information exist on secondary metabolite profiles of the section *Aspergillus* species isolated from animal feeds in Argentina and, to our knowledge, from any other country in the world. The secondary metabolite profiling of the 20 isolates belonging to the five most predominant section *Aspergillus* species contaminating different animal feeds revealed that, on the one hand, the five species produce a shared common metabolite profile. All the species were able to produce, under the experimental culture conditions used, cladosporin, echinulin, and neoechinulin A and B. However, variations in the secondary metabolite profile exist between the isolates of the same species. Only in the case of *A. montevidensis* the three isolates tested were all positive for these four secondary metabolites. In addition, preechinulin production was detected in one *A. montevidensis* and *A. chevalieri* isolates and physcion in two *A. chevalieri* isolates.

Production of auroglaucins, flavoglaucins and anthraquinones, such as physcion, catenarin, questin and questinol, has been reported before for *A. montevidensis*, *A. chevalieri*, *A. pseudoglaucus. A. ruber*, *A. glaucus* and *A. proliferans* [7]. None of these compounds, except physcion, was detected in the studied isolates. In addition, *A. montevidensis* and *A. ruber* isolates have also been reported to produce epiheveadride [15]. We did not detect this secondary metabolite in any of the studied isolates. On the other hand, all three of our *A. montevidensis* isolates and two of four *A. ruber* isolates tested positive for cladosporin, a compound that was not detected in the given species by Slack *et al.* [15].

Our secondary metabolite profiling results demonstrate that the *Aspergillus* section *Aspergillus* species isolated from different matrices and geographical origins do show different toxinogenic potential. All the five main species contaminating the animal feeds under study in Argentina had a consistent but, at the same time, rather limited secondary metabolite profile, at least under the culture conditions tested. No significant variation exists between species, though minor intraspecies isolate variations were detected. However, all the isolates were capable of producing echinulin, the precise secondary metabolite demonstrated to have serious mycotoxic effects on pigs, mice, rabbits [26,52]. The highest toxinogenic potential was detected in *A. chevalieri* as this species, depending on the isolate, tested positive for altogether six secondary metabolites.

This is the first report on detailed taxonomic identification and secondary metabolite profiling of *Aspergillus* section *Aspergillus* fungi contaminating feeds and primary raw materials used for animal feeding in Argentina. So far, most studies have observed additive or synergistic effects between consumption of different potent mycotoxins [71,72,73]. However, the effects of chronic simultaneous consumption of sub-toxic concentrations of fungal metabolites of lower toxicogenic potential, like the ones produced by the section *Aspergillus*, on animal or human health are currently poorly studied. Neither it is known if these compounds show additive or synergistic effects with each other or with other more hazardous mycotoxins. Therefore, further studies are needed to determine the full toxicogenic potential of *Aspergillus* section *Aspergillus* species under variable growth conditions to assess mycotoxicological risk that these xerophilic fungi can generate for animal and human health as feed and food contaminants.

## 4. Experimental Section

### 4.1. Samples

The teleomorphs of *Aspergillus* section *Aspergillus* were isolated from animal feeds and primary raw materials destined to rabbit, chinchilla and rainbow trout production in Buenos Aires, Córdoba, La Pampa, La Rioja, Mendoza, Rio Negro and Neuquén provinces in Argentina. Three different preparations of the rabbit, three of the chinchilla and two of the rainbow trout feeds were used for the isolation assays. The raw materials analysed consisted of alfalfa pellets, wheat derivatives (wheat millrun and wheat bran), bone and meat flour, soybean derivatives (pelleted soybeans and heat-inactivated soy) and corn derivatives (corn seeds and milled corn). Both the feeds and the primary raw materials studied included commercial products and animal producer fabricated non-commercial preparations. The fungal isolates obtained were maintained on solid CY20S medium at 4 °C and preserved on 18% glycerol stocks at −20 °C.

All the representative isolates described in this study are available upon request and will be accessioned in an international culture collection abroad.

### 4.2. Isolation of the Xerophilic Fungi

The initial isolation of xerophilic fungi and the genus level identification of *Aspergillus*, section *Aspergillus* isolates were carried out on dichloran 18% glycerol agar, DG18 [74]. The animal feeds and primary raw materials were processed according to Greco *et al.* [36] the serial diluted samples were inoculated on the selection medium and incubated at 25 °C for 7 days. In total, 21 samples from three different rabbit feeds, 25 from three chinchilla feeds, 28 from two rainbow trout feeds and one sample of each from the nine primary material types were analysed. The presence of section *Aspergillus* fungi was evaluated based on the growth morphology characteristics and isolated for further analyses. The isolation frequency (Fr%) of *Aspergillus* section *Aspergillus* was calculated for each starting material according to González *et al.* [75] with some modifications:

Fr% = number of samples with the sectionAspergillus/total number of samples × 100

### 4.3. Species Level Taxonomic Identification Based on Morphological Characters

The fungal isolates identified as members of *Aspergillus* section *Aspergillus* spp. in the initial xerophilic screen were used for further morphological species level identification according to Pitt and Hocking [8]. The isolates were inoculated in three points on CYA, MEA, GN25 and CY20S media and incubated at 5, 25 and 37 °C for 7 days. The colony characteristics were recorded after the incubation periods. The CY20S plates were incubated at 25 °C for 7 days further to allow ascospore development. The microscopic ascospore characteristics were observed and the sizes measured from lactophenol cotton blue stained samples using Nikon Eclipse E200 light microscope. The ability of the strains to grow on CY20S at 37 °C for 7 days was also evaluated [42]. The isolation frequencies (Fr%) of the five identified section *Aspergillus* species (*A. montevidensis*, *A. chevalieri*, *A. pseudoglaucus*, *A. ruber* and *A. proliferans*) were calculated for each starting material according to González *et al.* [75] as follows:

(2) Fr% = number of samples with the species/total number of samples × 100

### 4.4. Scanning Electron Microscopy (SEM)

For scanning electron microscopy analysis, the mature cleistothecia were harvested from fungal colonies grown for two weeks on CY20S and fixed in 0.1 M cacodylate buffer with 1% glutaraldehyde and 0.4% formaldehyde at room temperature [7]. The SEM service was purchased from Centro Científico Tecnológico CONICET Bahía Blanca (Argentina).

### 4.5. Taxonomic Identification Based on DNA Sequences

For mycelium harvest, fungi were grown for 7 days at 25 °C on solid CY20S plates. The mycelia were collected by scraping the surface of the plates and stored at −80 °C until use. The genomic DNA was extracted from 100–150 mg of mycelia manually grounded in a mortar with liquid nitrogen and the using DNeasy Plant Mini Kit (Qiagen, Intl.) according to manufacturer’s protocol. The quantification of genomic DNA was done with the fluorometer Qubit 2.0 (Life Technologies, Intl.).

The internal transcribed spacer (ITS) of nuclear ribosomal DNA was amplified using the primers ITS1 (5'-TCCGTAGGTGAACCTGCGG-3') and ITS4 (5'-TCCTCCGCTTATTGATATGC-3') [76]. The beta-tubulin gene fragment was amplified using the primers bt2a (5'-GGTAACCAAAT CGGTGCTGCTTTC-3') and bt2b (5'-ACCCTCAGTGTAGTGACCCTTGGC-3') [77]. The reaction volume used in PCR amplification was 40 µL and contained 1X of Taq buffer (Fermentas, Intl.), 1 µM of each primer (GBT Oligos, Buenos Aires), 0.2 mM of dNTPs (Fermentas, Intl.), 1.5 mM of MgCl_2_ (Fermentas, Intl.), 1 U of Taq DNA polymerase (Fermentas, Intl.) and 2 µL of genomic DNA template (3–15 ng/µL). The PCR amplification protocol for both primer pairs consisted of an initial denaturation step of 3 min at 94 °C, followed by 35 cycles of denaturation at 94 °C for 30 s, annealing at 50 °C for 30 s, extension at 72 °C for 1 min and a final extension step at 72 °C for 5 min. The PCR reactions were performed in a T-Personal thermocycler (Biometra, GmbH, Göttingen, Germany). The amplification products were separated by electrophoresis in a 1% agarose gel, stained with ethidium bromide, visualized with a UV transilluminator and documented with a Kodak Digital Science 1D system. Sequencing of the PCR amplicons was done with ITS1 and bt2a primers at Macrogen Inc. (Seoul, Korea).

### 4.6. Phylogenetic Analyses

Phylogenetic analyses based on the ITS and beta-tubulin sequences were run using the MEGA 6 program package [78]. The *Aspergillus* section *Aspergillus* type-strain reference sequences of altogether 18 species used in the analyses were obtained from GenBank (http://www.ncbi.nlm.nih.gov/genbank/). The species strain numbers, and the ITS and beta-tubulin sequences with their respective GenBank accession numbers are indicated in Supplementary Materials in Table A1. (Additional information of the strains and their former taxonomic nomenclature is available at Supplementary Materials in Table A2. The sequences of *Penicillium steckii*, strain NRRL 35625, were used as outgroup in the analyses. The isolate nucleotide sequences were visually inspected and aligned together with the reference sequences using the Muscle method [79]. The Maximum Likelihood analyses on the ITS, beta-tubulin and the concatenated sequences were run with Kimura 2 parameters, using Gamma distribution model and treating gaps, and missing data as partial deletion. The ITS alignment consisted of 567, the beta-tubulin alignment of 376 and the concatenated alignment of 943 nucleotide positions. The bootstrap values were generated with 1000 replicates. Sequence similarity percentages was calculated with the Muscle method.

### 4.7. Secondary Metabolite Profiles

Fungal growth and metabolite screening was carried out according to Slack *et al.* [15] with some modifications. The isolates were inoculated on CY20S and plates were incubated at 25 °C for 7 days to obtain heavily sporulating cultures. Spore inoculums were prepared in sterile dH_2_O and used for inoculating Erlenmeyer flasks containing 100 mL of CY broth without 20% sucrose at a final concentration of 1 × 10^4^ spores/mL. Static cultures were incubated in the dark at 25 °C for two weeks. After incubation, fungal cultures were filtered and a volume of 50 mL of crude filtrate was extracted twice with equal volumes of ethyl acetate in a 250 mL separatory funnel, concentrated under vacuum at 45 °C and 90 rpm, and dried under nitrogen gas. The residue was redissolved in 4 mL of methanol, filtered through a syringe filter (Acrodisc CR PTFE 0.45 µm, Gelman, USA) and analysed by HPLC-MS according to Nielsen and Smedsgaard [80]. The HPLC-MS system consisted of a Thermo Electron Corporation “Surveyor” equipped with a quaternary pump, an autosampler, ion trap (3D) (LCQ Advantage Max, Thermo Electron Corp., USA). The MS system was operated in the positive electrospray ionization (ESI) mode, N_2_ flow 10 L/min, voltage of 4.00 V positive, full scan over the mass range *m/z* 210–900 (single MS). The analytical column was a Thermo Scientific 50 × 2.1 mm 3 µm particle size. The injection volume was 10 µL. The analytical separation was performed using gradient elution with water (A) and acetonitrile (B) mobile phase, both modified with 0.1% formic acid. The following gradient was used: 85% A–15%B (initial), 0%–100% B (40 min), 100% B (5 min), 85% A–15% B (7 min), 85% A–15% B (2 min). The flow rate was 0.3 mL/min.

## Appendixes

**Table A1:** ITS and beta-tubulin sequences used in the phylogenetic analyses.

Species Name	Strain Number	GenBank Accession Number		Reference
		ITS	Beta Tubulin	
*A. montevidensis*	NRRL 108 ^T^	EF652077	EF651898	[64]
	NRRL 35697	EF652084	EF651902	[64]
*A. chevalieri*	NRRL 78 ^T^	EF652068	EF651911	[64]
	NRRL 4755	EF652071	EF651913	[64]
*A. cristatus*	NRRL 4222 ^T^	EF652078	EF651914	[64]
*A. intermedius*	NRRL 82 ^T^	EF652074	EF651892	[64]
*A. costiformis*	CBS 101749 ^T^	HE615136	HE801320	[42]
*A. glaucus*	NRRL 116 ^T^	EF652052	EF651887	[64]
	NRRL 120	EF652054	EF651889	[64]
*A. niveoglaucus*	NRRL 127 ^T^	EF652058	EF651905	[64]
*A. proliferans*	NRRL 1908 ^T^	EF652064	EF651891	[64]
	NRRL 114	EF652051	EF651886	[64]
	CCF 4146	HE578067	HE578076	[42,81]
**	IRAN 2088C ^TT^	KC473922	KC473925	[48]
**	IRAN 2089C	KC473923	KC473926	[48]
*A. brunneus*	NRRL 131 ^T^	EF652060	EF651907	[64]
*A. neocarnoyi*	NRRL 126 ^T^	EF652057	EF651903	[64]
*A. appendiculatus*	CBS 374.75 ^T^	HE615132	HE801333	[42]
*A. cibarius*	KACC 46346 ^T^	JQ918177	JQ918180	[18]
*A. pseudoglaucus*	NRRL 40 ^T^	EF652050	EF651917	[64]
	NRRL 17	EF652049	EF651916	[64]
*A. ruber*	NRRL 52 ^T^	EF652066	EF651920	[64]
	NRRL 76	EF652067	EF651921	[64]
*A. tonophilus*	NRRL 5124 ^T^	EF652081	EF651919	[64]
*A. cumulatus*	KACC 47316 ^T^	KF928303	KF928297	[19]
*A. xerophilus*	NRRL 6131 ^T^	EF652085	EF651923	[64]
*A. leucocarpus*	NRRL 3497 ^T^	EF652087	EF651925	[64]
*Penicillium steckii*	NRRL 35625	EF200085	EF198551	[82]

^T^ Ex-type strain, ^TT^ Ex-teleotype strain.

**Table A2:** Extra information on the previous and actual nomenclature of the species and strains of *Aspergillus* section *Aspergillus*, used in the phylogenetic analyses.

Actual Species Name	Strain Number	Previous Nomenclature
*A. montevidensis*	NRRL 108 ^T^	*E. amstelodami* (Mangin)
**	NRRL 35697	*E. amstelodami* (Mangin)
*A. chevalieri*	NRRL 78 ^T^	*E. chevalieri* (Mangin)
	NRRL 4755	*E. chevalieri* (Mangin)
*A. cristatus*	NRRL 4222 ^T^	*E. cristatum* (Raper & Fennell) Malloch & Cain
*A. intermedius*	NRRL 82 ^T^	*E. intermedium* (Blaser)
*A. costiformis*	CBS 101749 ^T^	*A. costiformis*
*A. glaucus*	NRRL 116 ^T^	*E. herbariorum* (Link)
	NRRL 120	*E. umbrosum*
*A. niveoglaucus*	NRRL 127 ^T^	*E. niveoglaucum*
*A. proliferans*	NRRL 1908 ^T^	*A. proliferans*
**	NRRL 114	*E. herbariorum **
**	CCF 4146	*A. proliferans*
**	IRAN 2088C ^TT^	*A. proliferans*
**	IRAN 2089C	*A. proliferans*
*A. brunneus*	NRRL 131 ^T^	*E. echinulatum*
*A. neocarnoyi*	NRRL 126 ^T^	*E. carnoyi*
*A. appendiculatus*	CBS 374.75 ^T^	*A. appendiculatus*
*A. cibarius*	KACC 46346 ^T^	*A. cibarius*
*A. pseudoglaucus*	NRRL 40 ^T^	*E. pseudoglaucum*
**	NRRL 17	*E. repens*
*A. ruber*	NRRL 52 ^T^	*E. rubrum*
	NRRL 76	*E. rubrum*
*A. tonophilus*	NRRL 5124 ^T^	*E. tonophilum*
*A. cumulatus*	KACC 47316 ^T^	*---*
*A. xerophilus*	NRRL 6131 ^T^	*E. xerophilum*
*A. leucocarpus*	NRRL 3497 ^T^	*E. leucocarpum*

* Misidentified strain. According to Hubka *et al.* [42] study, the correct name of the strain is *A. proliferans.*^T^ Ex-type strain, ^TT^ Ex-teleotype strain.
